# Plant Viral Vectors for Vaccine Development

**DOI:** 10.3390/vaccines14010081

**Published:** 2026-01-12

**Authors:** Mehdi Shahgolzari, Afagh Yavari, Srividhya Venkataraman, Mehrin Faija, Kathleen Hefferon

**Affiliations:** 1 Department of Medical Nanotechnology, School of Medicine, Zanjan University of Medical Sciences, Zanjan P.O. Box 45139-56184, Iran; mehdi.shahgolzari@gmail.com; 2 Department of Biology, Payame Noor University, Tehran P.O. Box 19395-3697, Iran; yavari.afagh@pnu.ac.ir; 3 Independent Researcher, Toronto, ON L5M 6K5, Canada; byokem@hotmail.com; 4 Virology Laboratory, Department of Cell & Systems Biology, University of Toronto, 25 Willcocks Street, Toronto, ON M5S 3B2, Canada; muf2@cornell.edu

**Keywords:** expression vectors, plant viruses, vaccine

## Abstract

Plant viruses are useful tools for quickly and easily producing recombinant proteins in plants. Compared to systems that use genetically modified plants, viral vectors are easier to work with and can produce recombinant proteins faster and in larger amounts. Recently, there has been growing interest in using plant viruses as vectors to make vaccines, either as whole proteins or as small parts displayed on plant virus particles. The best examples for this purpose are tobacco mosaic virus, cowpea mosaic virus and potato virus X. Vaccines made using these viruses target various human and animal diseases and have often triggered immune responses and provided protection against infections. This review looks at the benefits of using plant virus vectors, the progress in developing different viral vector systems, and immune studies that support the idea of vaccines made from plant viruses.

## 1. Introduction

Vaccinology started in 1789 with Edward Jenner’s smallpox vaccine with pivotal disease prevention [1,2]. In the 19th century, Louis Pasteur contributed to the field by creating vaccines through pathogen attenuation [3]. Cell culture techniques developed by the mid-20th century led to the creation of inactivated and attenuated vaccines, which played a crucial role in eradicating outbreak diseases and saving millions of lives [2]. Before the late 20th century, live attenuated, toxoid, and subunit vaccines were used as conventional vaccines, prior to the emergence of molecular biology and significantly reduced the prevalence and severity of diseases [4]. Despite ongoing research for decades, effective vaccines for specific human pathogens are still lacking due to challenges such as high genetic variability, persistent or latent infections, and the difficulty in eliciting sterilizing immunity [4,5]. Furthermore, traditional vaccines face major limitations, such as safety risks from the reversion of live attenuated viruses, contamination with live organisms, and potential adverse effects in immunocompromised individuals [4]. Their production is also expensive, time-consuming, and requires high biosafety standards along with specialized laboratory facilities [4,6].

Recent advancements in genetic engineering, immunology, structural biology, and nanotechnology have led to the emergence of innovative vaccine platforms. These innovations encompass a range of cutting-edge technologies in the field of vaccines, including recombinant viral vectors, virus-like particles (VLPs, viral capsids lacking genomes), mRNA vaccines, synthetic DNA vaccines, and bacterial vector vaccines [4]. Such technologies provide significant advantages over traditional vaccines, facilitating rapid and efficient responses to new pathogens and pandemics. Recombinant plant viral vectors are highlighted as a promising platform among various technologies. The use of plant viruses in vaccine development can rely on genome capability for encode foreign antigens, intrinsic adjuvant properties for eliciting innate immune responses, and safety. Furthermore, they can also be engineered for targeted antigen delivery to specific cells or tissues. Plant virus expression vectors enable rapid, large-scale gene expression without the need for extensive plant transformation, often achieving substantial levels within days. Increasing the number of host plants can enhance scale-up of protein expression Furthermore, facile steps or just partial purification can be applied for isolation and purification of vaccine proteins [7]. These features enable plant virus expression vectors to serve as versatile tools for combating infectious diseases today, particularly in developing countries.

## 2. Plants as Biofactories in Vaccine Development

Vaccines can be produced in various host systems, including bacteria, yeast, insect cells, and mammalian cells [8]. Advancements in molecular biology in the 1980s facilitated the creation of subunit vaccines through the utilization of proteins sourced from pathogens. While mammalian cell systems are commonly used for vaccine production, vaccine production poses safety risks due to potential toxins and infectious agents, as microbial contamination can remain undetected post-purification, complicating quality control during large-scale manufacturing [9,10]. *E. coli* is deficient in key post-translational modifications, and while yeast and insect cells present some alternatives, their immunological disparities restrict their effectiveness.

From the late 1980s, plants have been suggested as a promising platform for recombinant protein production [11]. Today, producing biopharmaceuticals (drugs and vaccines) in plants using molecular biology techniques is known by the term ‘molecular farming’ [12]. Plant-based platforms for vaccine production provide various benefits compared to conventional expression systems. First, plant-made vaccines are significantly cost-effective, with production costs potentially up to 1000 times lower than mammalian cell-based systems and 50 times less than *E. coli* bioreactors in terms of protein yields [13]. For example, the Bean Yellow Dwarf Virus (*BeYDV*) system yielded antibodies, producing 1.2–1.4 g/kg of fresh weight that effectively neutralized Zika virus and other flaviviruses, thus showing the capability of the BeYDV system to rapidly and inexpensively produce a wide range of multi-protein pharmaceutical complexes, then illustrating its potential for aiding in the development of therapeutic solutions [14]. Plants utilize sunlight, carbon dioxide, water, and nutrients in photosynthesis to produce materials and energy. This not only lowers production costs but also improves scalability, with a larger cultivation area directly increasing output [15]. Second, plants are a safer option for human and veterinary applications than mammalian cell systems because they do not harbor animal pathogens like microbes or prions [16,17]. Third, eukaryotic post-translational modifications, including glycosylation and the formation of disulfide bonds, that are critical for maintaining the biological function and structural integrity of viral vaccines, can be achieved using plant-based expression systems [15,18]. Fourth, plant-based platforms provide remarkable scalability, allowing quick production capacity expansion and significantly reducing manufacturing timelines, which is vital for addressing infectious disease outbreaks and public health emergencies [19,20]. Lastly, plant-based vaccines, as edible vaccines, can be expressed in the edible parts (e.g., seeds, leaves, tubers, fruits, and vegetables) of plants [12,15,21]. Thus, plants have become a compelling medium for producing vaccines. Stable transformation of the plant chloroplast genome and that of the nuclear genome as well as viral transient expression are available strategies for vaccine production in plants.

## 3. Plant Viral Transient Expression in Vaccine Development

Since the 1980s, plant viruses have served as a vehicle for introducing foreign genetic material into plants. With continuing advancements in virology and molecular biology techniques, improved expression systems have been developed with quick, high-level expression of transgenes, and improved containment—especially in movement-defective systems that prevent both vertical and horizontal gene transfer [22]. Structural flexibility of plant viruses and genetic diversity make them ideal platforms for designing customized tools for biotechnological applications. These viruses can enhance immune responses more effectively than conventional vaccines, highlighting their potential in vaccine development [23]. The ability of plant viruses to present multiple antigens leads to strong immune responses, proving valuable for next-generation vaccines with improved immunogenicity and efficacy [24,25]. Plant viruses and plant virus-like particles (pVLPs) are not only significant in vaccinology but also serve as effective vehicles for drug delivery, capable of encapsulating therapeutic molecules and delivering them directly to target cells, which reduces off-target effects and improves therapeutic results [26,27]. Another method employs plant viruses as adjuvants in vaccine development, enhancing safety by not replicating in animal cells. In contrast, plant viral-based vaccines typically do not require additional adjuvants, simplifying their composition and formulation compared to other vaccines.

Plant viruses are effective in producing recombinant proteins. They enable large-scale synthesis of heterologous, expensive proteins such as antibodies and vaccines. As cellular parasites, they can produce a variety of viral proteins while precluding post-transcriptional gene silencing (PTGS) and degradation of heterologous proteins, thereby facilitating systemic infection in plants. Plant viral vectors offer benefits as an alternative to conventional transgenic systems, including faster expression, higher yield production, cost and time savings, and adaptability for high throughput and scalability.

The tobacco mosaic virus (TMV) is credited with founding virology and has significantly influenced various fields, including biotechnology, evolution, nanotechnology, and plant virology [28]. Much evidence supports the notion that plant viruses can be utilized to produce recombinant proteins in plants. Plant viral vectors, initially a proof-of-concept idea, have evolved into a cost-effective tool for producing heterologous proteins like vaccines and antibodies (Table 1). Plant viruses are cellular parasites and exhibit several properties: (1) they reproduce their genome and protein components through the use of host cell machinery; (2) they have evolved strategies for silencing the PTGS of the host and suppressing protein degradation; and (3) they can establish systemic infection throughout the entire plant. Thus, using these properties of plant viruses, plant viral vectors offer significant advantages over traditional transgenic and chloroplast (transplastomic) expression systems: (1) faster expression and higher yield production; (2) time and expense savings; and (3) a system with high throughput, flexibility, and scalability [29]. Over the past 30 years, a variety of expression techniques have been used to create potent viral vectors [22].

## 4. Plant Virus Vector–Based Strategies in Vaccine Development

**Figure 1:**
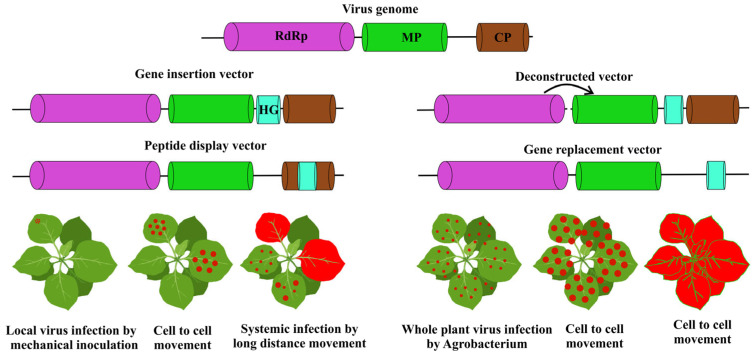
Plant viral vectors and expression strategies. The viral replicase, movement protein (MP), coat protein (CP), and heterologous gene (HG).

Molecular biology techniques such as RNA template-based cDNA, particularly ex-pression systems in single-stranded RNA viruses common among plant viruses, have led to advancements in vaccinology [22]. Moreover, *Agrobacterium tumefaciens* has aided in facilitating viral infection [15,35,36]. Commonly, viral infections occur by mechanical inoculation of plant leaves, or by nucleic acid transfection. The process of agro-infection makes use of *Agrobacterium tumefaciens* to efficaciously deliver cDNA into plant cell nuclei. After the cDNA is transcribed and processed in the host nucleus, the cDNA in the plant expression cassette creates a replicating nucleic acid that causes infection [15]. Moreover, agro-infection provides opportunities to use viruses that cannot be mechanically transmitted in nature, where the only requirement for infection is a natural insect vector for the virus. Viral vectors that can be used include peptide display vectors, modular vector systems, gene insertion vectors, and gene substitution vectors (Figure 1).

### 4.1. Gene Insertion Vectors

Gene insertion vectors are constituted of modified versions of viruses that have an added open reading frame (ORF) coding for the desired protein to be expressed [22,37]. The target protein can either be fused to the coat protein (CP) of the virus or expressed independent of the CP [15,22,38]. TMV and potato virus are notable vectors, both featuring single-stranded positive-strand RNA genomes [22,38]. Insertion of the chloramphenicol acetyltransferase gene (CAT) gene between the motor protein and CP genes enables TMV to replicate, form subgenomic RNAs, assemble accurately, and express the intended gene activity [39,40]. A viral expression vector derived from potato virus X (PVX) was created, which incorporates a bacterial β-glucuronidase gene. The vector, pGC3, successfully infected *Nicotiana clevelandii* and *N. tabacum*, demonstrating GUS expression in the leaves [41]. A common vector is one derived from cowpea mosaic virus (CPMV). Inoculation of cowpea with the CPMV/S-2A- green fluorescent protein (GFP) construct, fused to jellyfish green fluorescent protein, led to systemic infection within 10 days and yielded approximately 1% of total soluble protein as recombinant GFP [42]. However, the use of the subgenomic promoter upon amplification led to homologous recombination resulting in instability of the vector and loss of the foreign gene [40]. The first generation of viral vectors has revealed issues, notably the risk of complete viruses disseminating systemically in host plants, which raises biosafety and biosecurity concerns. Large exogenous inserts can impair virus assembly and the infection area, whereas a cleavage site may lead to a combination of free and fused proteins. Promoter duplication can result in homologous recombination of repeating sequences, leading to instability in constructs, despite significant attempts to address these challenges [15].

### 4.2. Gene Replacement Vectors

In gene replacement or substitution vectors, the endogenous viral sequence is substituted with a heterologous gene (HG) or genes identified in another organism [22]. A gene replacement vector is created by substituting an endogenous viral sequence with a gene of interest (GOI). The viral genome lacks certain essential elements required for protein expression, including the CP that in the majority of viruses is a necessity for systemic movement. The removal of CP allows for the effective elimination of bio-contamination threats, as it is no longer necessary for systemic movement. Additionally, viral vectors can accommodate larger inserts when the viral CP is substituted with a gene of interest, offering advantages over traditional gene insertion vectors. Musiychuk et al. created pBID4, a launch vector designed to increase the expression, immunogenicity, and stability of modified lichenase and its fusion products with proteins like plague, anthrax, and influenza [43]. For example, two hemagglutinin (HA) antigens from influenza A/Vietnam/04 (H5N1)—LicKM-H5GD and LicKM-H5SD—were expressed in *Nicotiana benthamiana*, purified to >80% by affinity chromatography, and shown to bind specifically to antibodies in sheep sera raised against HA [22,43]. Moreover, using vectors that contain the full viral genome along with a gene of interest (GOI) can generate large plasmids, limiting efficiency and flexibility.

### 4.3. Modular or Deconstructed Vectors

Modular or deconstructed vectors represent an advanced category of viral expression system, which emerged from an understanding that not every component in the overall virus is critical to express the target protein(s) properly. Viruses may be broken up into their constituent parts (genomic elements) that function together during the process of infection, resembling multipartite viruses. Moreover, agro-infection allows co-delivery of multiple components [22]. The modular system fragments the viral genome to form provectors with essential elements such as RNA-dependent RNA polymerase (RdRp) to improve protein expression [44,45]. To establish an integrated system, provectors should be agro-infiltrated into a single cell, and co-expressed. In a modular system, viral components are divided and put into binary vectors derived from agrobacterium that are mixed and co-infiltrated into the leaves of the plant. By mixing these two components, the replicon size is reduced because of transgene insertion, while allowing for the other viral components to be injected into the cell during infection. Currently, one of the most utilized vectors is the TMV-based deconstructed system created by Icon Genetics (Halle, Germany), and today part of Bayer Innovation GmbH. In *N. benthamiana*, this includes deconstructed viral vectors like pEAQ vectors [46], Lindbo’s TRBO [47], geminiviral vectors from Mason’s group [14,48], and magnICON^®^ systems (NOMAD Biosciences, Berlin, Germany) [49,50], likely enhanced with humanized glycosylation approaches.

These integrations enable quick accretion of proteins and complex glycoengineering that are critical for the generation of therapeutics of high quality. Wang et al. developed two chimeric virus vectors that were based on the pepper mild mottle virus (PMMoV) and the pod pepper vein yellows virus (PoPeVYV). These constructs upon delivery through agrobacterium-based infiltration resulted in successful biosynthesis of the mGFP-HBsAg fusion protein using PoPeVYV, while there occurred weak expression of this protein using the PMMoV, which suggests vector-dependent selectivity in plant-based HBsAg expression systems [51].

### 4.4. Peptide Display Vectors

This technique applies the strategy of fusing the target gene with a viral coat protein (CP) gene to allow surface display on chimeric virus particles (CVPs) [22,52]. Foreign peptides can be attached to the N- or C-terminus of the CP, or indirectly by adding additional sequences, such as a translational readthrough context [53] or an F2A sequence [54]. The viral functions of the virus, including replication, RNA and protein synthesis, and self-assembly of particles, should not be impaired with the fused CP gene. While these functions often limit the maximum size of insertable sequences, many researchers have successfully employed peptide display systems as early from as 1986 and continue to use this technology in the commercialization of plant-derived vaccines [55,56]. Subunit vaccines use small peptide or protein epitopes, but their limited size hinders recognition by antigen-presenting cells, resulting in weak or incomplete immune responses unless presented on carriers or with adjuvants [57,58]. Plant viruses by enlarging epitope size can enhance identification and immune responses [59,60]. Additionally, plant viruses are confined to their host-plant range and lack the ability to replicate or cause disease in animals, ensuring biosafety for vaccine and therapeutic applications [61]. Lico et al. produced the nucleoprotein (NP)-displaying chimeric virus particles (NP-CVPs) via incorporation of an influenza A virus NP sequence into the N-terminus of PVX [62]. Foreign peptides fused with PVX CP were displayed on NP-CVPs, yielding ~1.1 mg/g in *N. benthamiana*. Immunized mice produced antigen-specific CD8+ T-cells without adjuvants, indicating CVPs can activate cell-mediated immunity and serve as effective vaccine platforms.

## 5. Type of Plant Viral Vectors

### 5.1. Comovirus (Cowpea Mosaic Virus (CPMV))

CPMV-derived expression vectors for peptide display were initially developed in the early 1990s [63]. In this system, the surface of the CPMV vectors displays small antigenic peptides (epitopes). Cowpea CPMV features a highly ordered, three-dimensional icosahedral symmetry, allowing repetitive display of multiple epitope copies on its surface, which enhances immunogenicity and establishes it as an effective vaccine carrier platform [15,64]. CPMV achieves high yields in plants, producing 0.5 to 2 mg purified virus per gram of fresh leaf tissue. Its high titer, combined with non-pathogenicity in mammalian cells, makes CPMV an ideal epitope presentation platform for vaccines, balancing both biosafety and yield effectively [15,65]. CPs of CPMV have three major sites for epitope insertion or insertion of peptide-specific sequences. The most commonly used location for insertion is the βB–βC loop present on the small CP (SCP). However, in addition to this site, the βC′–βC″ loop located on the SCP and the βE–αB loop located on the large CP (LCP) are available for inserting epitopes [15,66]. CPMV has also been employed in various gene expression strategies, including using it as a gene replacement vector, gene insertion vector, deconstructed vector, and in combination with other viral vector systems [15,67].

### 5.2. Tobamovirus (Tobacco Mosaic Virus (TMV))

The *Tobamovirus* genus in the family Virgaviridae includes rigid, monopartite, rod-shaped plant viruses with a positive-stranded RNA genome. The N-terminus, C-terminus, and a 60s loop are exposed on the viral surface enabling potential bioconjugations [68]. Furthermore, TMV’s stability, modifiability, and self-assembly make it ideal for peptide display [69]. In a pioneering study, by using TMV as a template for molecular farming, researchers successfully engineered TMV to present the type 3 poliovirus epitope showcasing its utility as a peptide display platform [55]. Turpen et al. used TMV to display *Plasmodium* epitopes, producing malarial subunit vaccines in tobacco plants [70]. The G5-24 epitope derived from the rabies virus glycoprotein and the 5B19 epitope derived from the mouse hepatitis virus spike glycoprotein were efficiently displayed on the surface of the TMV particles [69]. Takamatsu et al. replaced TMV’s coat protein gene with a bacterial CAT gene [71].

**Figure 2:**
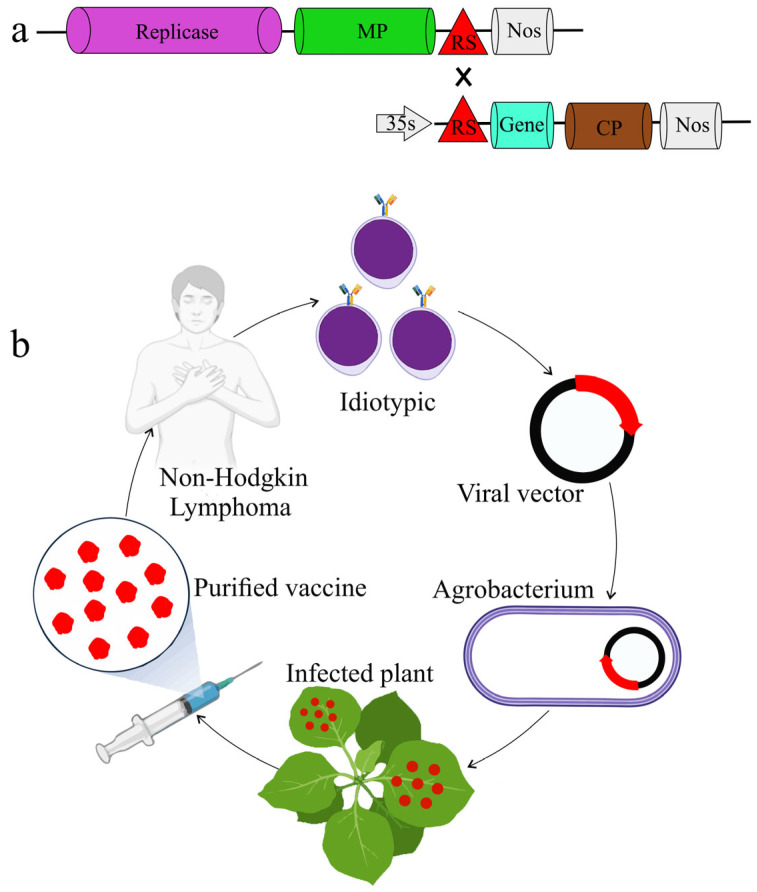
Deconstructed TMV expression system. (**a**) Deconstructed TMV-based expression vector designed for the production of active replicons in plant cells. (**b**) The TMV expression system in developing a personalized vaccine for Non-Hodgkin Lymphoma (NHL). From patients with Non-Hodgkin Lymphoma (NHL), proliferating B-cells expressing a distinct idiotype are identified. A viral vector is created by cloning cDNA with an idiotypic region. Agrobacterium is created from the viral vector that contains the idiotype cDNA. Whole plants are infected with agrobacterium that carries a viral vector. Plants are harvested for their idiotypes that carry vaccine proteins. To induce an immunological response to cells expressing the idiotype, the original patient is infected with purified vaccination protein.

The genome of TMV has been engineered to generate a ‘deconstructed’ form made of two separate modules (Figure 2a). One module harbors the sequences that are essential for replication, while the other contains cassettes to insert foreign genes. This enables biopharmaceutical proteins to be expressed at high levels in plants using this viral replicon system. For instance, deconstructed expression vectors derived from TMV are being explored as propitious therapeutic options to treat some types of cancer, such as Non-Hodgkin Lymphoma (NHL). Plants can serve as a source of degenerate B-cells by agro-infiltrating the vectors into the tobacco leaves, allowing for rapid and inexpensive expression of lymphoma vaccines, potentially acting as short-term therapy (Figure 2b) [72].

### 5.3. Potexvirus (Potato Virus X (PVX))

Several potexviruses have been used to engineer expression vectors, inclusive of *Alternanthera* mosaic virus (AltMV) [73,74], foxtail mosaic virus (FoMV) [75,76], cassava common mosaic virus (CsCMV) [77], *Cymbidium* mosaic virus (CymMV) [78], pepino mosaic virus (PepMV) [79,80], *Narcissus* mosaic virus (NMV) [81], *Zygocactus* X virus (ZVX) [82], *Plantago asiatica* mosaic virus (PlAMV) [83], potato virus X (PVX) [84], and bamboo mosaic virus (BaMV) [85,86,87]. Of these, PVX has been successfully used for generation of vaccine candidates against cancer and other diseases. The following section describes recently published findings on the potential applications of PVX-derived vectors as vaccine candidates.

Potato virus X (PVX), belongs to the *Potexvirus* genus with a single-stranded (+)-sense RNA genome that has a 3′ poly(A) tail and a 5′ cap structure. The genomic RNA is encapsidated within a protein shell composed of 1300 capsid protein subunits encoded by the virus. The PVX genome has 5 ORFs [84] that encode the replication protein (RNA-dependent RNA polymerase (RdRp)), three triple gene block (TGB) proteins that function as movement proteins, and the capsid protein that is necessary for the assembly, cell-to-cell transmission, and systemic movement of the virus.

Potato virus X PVX functions as an inimitable nano-scaffold for the development of vaccines by genetic engineering of the virus to display on its surface immunogenic peptides that can elicit a strong immune response. Scientists have genetically modified PVX to chemically conjugate or fuse foreign peptides to the PVX CP subunits to generate chimeric virus particles (CVPs). Such modified forms of the PVX particles can enable multivalent presentation of antigens, augmenting their capability to target B-cells and induce robust immune reactions in animal models. The filamentous, flexible structure of PVX makes it an effectual nanocarrier for antigen presentation to the immune system. Plants infected with the engineered constructs of PVX can generate high yields of the CVPs capable of displaying the desired antigens or peptides. The surface of the potato virus X–derived chimeric virus particles (PVX CVPs) are densely covered with several copies of the same antigenic peptide, inducing a strong immune response. Biodistribution analyses reveal that these PVX CVPs are well tolerated, in addition to displaying broad biodistribution and having high bioavailability in animal models. PVX antigen carriers exhibit a tropism for B-cells that are essential for antibody responses and immune memory. PVX provides a versatile platform enabling the display of various types of antigens and peptides for several vaccine applications.

The viral particles of PVX have a filamentous structure that allows them to transport large payloads that are advantageous for biomedical imaging since this needs the generation of multifunctional scaffolds with a high aspect ratio [88]. PVX is capable of superior homing into tumors and has better retention properties when compared to that of spherical nanoparticles. Since PVX is constituted of a protein-based nanoparticle, its unique functional properties, in addition to enhanced biocompatibility, render it more suitable for biomedical applications when compared to that of synthetic nanomaterials. Furthermore, PVX nanoparticles have diminished toxicity in vivo in addition to better pharmacokinetic profiles.

PVX vectors afford high-level expression of recombinant proteins, rapid transport and systemic infection within the plant. In plant systems, PVX has been employed as a full-length expression vector with capability to infect distal tissues. It has also been used as a deletion mutant vector in which genes important for systemic and cell-to-cell movement have been deleted. Many approaches have been undertaken to generate PVX-derived transient expression vectors.

These methodologies include the following: the employment of a double sub-genomic promoter to improve expression of exogenous genes [89]; foreign protein expression wherein the foreign gene is directly fused to the truncated CP N-terminus [60,90]; the CP ORF and the ORF encoding the antigen of interest separated by the FMDV 2A autocatalytic peptide such that a ribosomal skip mechanism can assist in the release of two separate peptides that originate from a single transcription unit [84]; expression of a bicistronic mRNA that is under the control of the CP subgenomic promoter in which the heterologous and coat protein genes are separated by an IRES (internal ribosome binding site) [85].

In a pioneering study, the pGR106 expression vector derived from PVX was used in the expression of the HPV L2 and E7 proteins in *N. benthamiana* [91]. The concentration of purified protein was 2.8–4.3 mg/100 g of fresh leaves. In an effort to generate vaccine prototypes, virus particles were produced wherein the HIV type I gp41 ELDKWA epitope was fused to the PVX CP N-terminus. Immunogenicity investigations in mice models showed that intraperitoneal or intranasal immunization of mice led to high expression levels of specific IgG and IgA antibodies for HIV even in the absence of adjuvants [60]. Čeřovská et al. (2012) generated a fusion protein composed of the HPV16 L2 minor capsid protein and the CP of PVX [92]. This generated a yield of 170 mg of this protein for every kg of fresh leaf tissue. Subcutaneously immunized test mice showed the elicitation of antibodies against both the L2 epitope and the PVX CP. The Influenza virus M2 protein and the trans-membrane domain which is highly conserved was fused with the Hepatitis B core antigen (HBc) and expression of this fusion protein using a PVX vector resulted in a yield of 2% of the TSP. The purified form of this protein was found to be immunogenic in mice wherein the immune reaction was Th1-polarized [93]. PVX expressing the HPV-16 E7 protein in *N. benthamiana* when administered along with the Quil A adjuvant was demonstrated as highly immunogenic in mice by eliciting both cell-mediated and antibody responses [94,95]. The IgG antibody isotypic profile revealed that both Th1 and Th2 immune responses took place and 40% of the mice continued to lack HPV tumors following challenge with a tumor cell line that expressed E.

Further, tumors developed in mice vaccinated with the genetically engineered plant-generated E7 were significantly reduced in volume in comparison with the tumors that appeared in untreated mice.

CVPs of PVX displaying the 2F5 linear epitope (2F5e) of the HIV-1 gp41 envelope protein could elicit expression of epitope-specific IgG and IgA, upon intranasal or intraperitoneal administration in normal mice models without the necessity for co-administered adjuvants.

Notably, pulsing of the human dendritic cells with these PVX CVPs led to the activation of a primary antibody response specific to 2F5e in acute combined immunodeficient mice engrafted with the lymphocytes [96].

PVX CVPs displaying the MHC class I-restricted Influenza virus peptide have been genetically engineered [62]. These CVPs were reportedly stable and triggered epitope-specific CD8+ T-cells while removing the requirement of adjuvant co-administration. PVX CVPs serve as better carriers of epitopes having adjuvant characteristics probably on account of their intricate particulate structure along with the presence of the PVX genomic RNA that may elicit a TLR-7 response on antigen-presenting cells [97].

Due to these inimitable features, PVX has the inherent capability to elicit whole immune responses. The CVPs of PVX have been engineered to display the fibronectin-binding protein D2 peptide from *Staphylococcus aureus* [98], two E2 glycoprotein peptides from the classical swine fever virus [59], and the HCV hypervariable region I R9 peptide [54].

PVX particles displaying the HER2 extracellular domain peptide have been generated by attaching the Lys residue on the PVX CP surface through a heterofunctional NHS-(poly)ethyleneglycol (PEG)4-maleimide linker [99].

The antigen HER2 is typically expressed on breast cancer cells wherein it induces B-cell activation and the production of antibodies. These PVX-based chemically derivatized particles were demonstrated to effectively elicit HER2 immunological tolerance in HER2-positive human cancer cells in mice models. PVX nanoparticles labeled with A647 upon bioconjugation with a 12-amino-acid peptide containing affinity to the EGF receptor (EFGR) are capable of identifying and imaging carcinoma cell lines upregulating EGFR and show a preference to partition to cancerous cells instead of macrophages [100]. Moreover, PVX particles that were conjugated to the HER2 peptide specific to the Herceptin (Trastuzumab) monoclonal antibody showed capability to stimulate the HER2-positive cell line apoptosis [101].

VNPs of PVX when functionalized with protein A fragments can be shown to display entire antibody molecules, since protein A will bind to the Fc region of the IgG heavy chain [102,103]. Such PVX-derived particles can serve as a plug-and-play mechanism capable of displaying an array of antibodies having a level of orientational control that is unattainable by chemical conjugation.

Diagnosis of Sjögren’s syndrome (pSjS), a chronic autoimmune disorder, can thus be hampered due to its heterogeneity [104]. The human autoantigen lipocalin-derived Lipo peptide when displayed on PVX VNPs functions as a nanoparticle system that showed specificity to pSjS patient sera having superior reactivity compared to the peptide by itself [105].

Deconstructed vectors derived from PVX have been variously used as expression systems. Efficient expression of the gene insert has been achieved by replacement of the TGB movement protein and CP genes [106]. PTGS suppression enhanced protein yields in which transiently co-expressed TBSV P19 or TEV HC-Pro, respectively, resulted in 44% and 83% augmentation of gene expression [45]. Such a gene-silencing suppression mechanism was also used to enhance the expression of a canine oral papillomavirus highly labile L1 vaccine protein fused to a chloroplast targeting peptide and expressed from a PVX vector [107].

### 5.4. Pepino Mosaic Virus (PepMV)

The widespread PepMV, a Potexvirus, is a propitious candidate for the expression of recombinant proteins in plants due to its high-level accretion in its hosts as well as the absence of acute infection symptoms. PepMV undergoes distribution into most plant parts, establishing infection in flowers and roots. These properties render this virus as an attractive system for use as a tool to silence plant genes in various tissues.

Sempere et al., in 2011, reported the generation of a PepMV-based vector that had the ability to establish systemic foreign protein expression in *N. benthamiana* plants [80]. Several strategies were used to enable stable expression of transgenes inclusive of substitution of the CP gene, CP subgenomic promoter duplication, and a translational fusion of genes. A stable PepMV vector was generated through expression of the transgene fused with the CP through the incorporation of the sequence encoding the catalytic peptide of the foot-and-mouth disease virus (FMDV) 2A s. Through this, they achieved high recombinant protein expression. Incorporation of an enhancer of translation into the PepMV-based vector assisted with increased foreign protein expression, with protein levels reaching 4% of total soluble protein [79] These expression levels illustrate that PepMV-based vectors have potential for the large-scale biosynthesis of pharmaceutical compounds.

### 5.5. Alfamovirus (Alfalfa Mosaic Virus (AlMV))

Alfalfa mosaic virus is prevalent worldwide and has a broad host range. It is the type member of the genus *Alfamovirus* and belongs to the family Bromoviridae. AMV spreads upon sowing of infected seeds or by aphid vectors [108]. AMV is a complex virus constituted by four distinct types of particles inclusive of three rod-shaped particles (30 to 57 nm) and one spherical particle. The rod-shaped particles have a hemispherical end containing pentagonal symmetry besides which there occurs a cylindrical portion having hexagonal extensions in different configurations. AMV harbors a single-stranded RNA genome constituted by RNA1, RNA2, and RNA3. Additionally, the subgenomic RNA4 is generated by transcription from the RNA3 negative-sense strand. Each of the genomic RNAs is encapsidated inside the rod-shaped particles in addition to RNA4. Each of RNA1 and RNA2 contains an ORF, respectively coding for the P1 and P2 subunits of the viral replicase. RNA3 has two ORFs from which movement proteins as well as capsid proteins are expressed. The translation products of AMV-CP or RNA4 are necessary for viral RNA replication, packaging, transmittance, and infection.

VLPs have been designed using AMV for use in vaccines because of their intrinsic structural stability and ability to elicit an immune response. This VLP can express foreign antigens in plants and deliver them via various plant viruses designed for treating common diseases, such as cancers and infections, through induction of antigen-specific antibody and T-cell responses. A major advantage is that it is a non-cytolytic immune modulator that can promote in situ vaccination due to its lack of a cytolic effect on immune system function [109]. It can induce an immune response to the viruses which have been designed experimentally and has been used directly in tumors to promote immunostimulation in the tumor immune microenvironment, leading to tumor growth and metastasis repression. AMV can effectively recruit immune cells and modulate cytokines, making it a strong candidate as a potential therapeutic vaccine. In addition to its potential as a vaccine, its inherent stability, safety due to no adverse effects on human pathogens, low cost (the initial plants used to produce AMV can be very economical), and the ability to mass produce it rapidly, further reinforce its potential as a therapeutic vaccine.

AMV constitutes a molecular platform capable of presenting foreign antigens towards initiating immune recognition against these antigens. In this context, a chimeric peptide having antigenic determinants of the nucleoprotein (N protein) and the glycoprotein (G protein) from rabies virus was amplified and cloned as an AlMV CP translational fusion product.

Upon one dose of classic rabies virus vaccine, inoculated mice demonstrated detectable levels of neutralizing antibody to rabies virus compared with control mice [110]. Furthermore, the Pfs25 protein expressed on the surfaces of *Plasmodium falciparum* gametes, ookinetes, and zygotes has been considered as a prime target for the development of malaria transmission-blocking vaccines. This Pfs25 malarial vaccine candidate has been engineered as a fusion with the AMV CP and expressed in plants [111]. This vaccine candidate named Pfs25 VLP-FhCMB has been manufactured at pilot scale in *N. benthamiana* plants as per GMP guidelines. Phase I and first-in-human study using healthy adult volunteers was performed in combination with the Alhydrogel^®^ (InvivoGen, San Diego, CA, USA) adjuvant wherein the plant-derived Pfs25-AMV VLP purified from the plants was injected into the subjects.

At doses examined in this investigation, the above vaccine formulation was shown to stimulate robust antibody reactions at doses over 30 mg total protein and proved to be safe with no occurrence of toxicity and adverse events associated with the vaccine, demonstrating that this vaccine had acceptable safety profile and tolerability.

AMV can carry small antigenic peptides as fusions with its coat proteins and induce antigen-specific immune response. *Bacillus anthracis* protective antigen domain-4 small loop 15 amino acid epitope (PA-D4s) was placed immediately after the first 25 amino acids at the N-terminus of AMV CP [112]. This retained the genomic activation and association of the CP to viral RNAs. The resultant recombinant AMV particles were efficaciously expressed in tobacco plants, allowing for facile purification and immunological analyses in addition to exhibiting systemic proliferation and extended stability in the plants. Mice which were injected intraperitoneally with recombinant AMV particles expressing the PA-D4s epitope demonstrated a distinctive immune response wherein the immunized mice sera could recognize both the AMV CP as well as the *Bacillus anthracis* PA antigen.

Shahgolzari et al. 2021 showed that AMV upon administration elicited various immune changes within the tumor microenvironment, leading to an efficacious immune response against the 4T1 murine triple-negative breast cancer model, which is highly aggressive, metastatic, and recalcitrant to treatment with conventional immunotherapy [109]. Mechanistic investigations showed that AMV ISV augments the production of inflammatory cytokines, immune effector cell infiltration, and costimulatory molecules, in addition to downregulating immune-suppressive molecules. In situ AMV vaccination switches the tumor microenvironment from one that is immunosuppressive to an immunostimulatory mode. AMV nanoparticles are neither cytolytic nor trigger apoptosis of the tumor cells in a direct manner but instead stimulate immune reactions against the tumor. Thus, AMV is naturally immunogenic, and this property can be used to develop AMV as an immunostimulatory agent for in situ vaccination into tumors. AMV ISV notably delays the growth of the tumors and tumor metastasis. The cytokines secreted by ISV with AMV provide direct evidence for its immunostimulatory properties, reflecting immune modulation mechanisms. AMV elicits more granulocytes inside the tumors and alters the tumor microenvironment through stimulating and recruiting immune-modulating cytokines, leading to phenotypic changes within the immune cells.

A chimeric peptide containing the rabies virus nucleoprotein and glycoprotein determinants was fused to the AMV CP N-terminus and cloned into an AMV-based vector. When this fusion protein was transfected into spinach and *N. benthamiana* plants, it led to high-level (0.4 mg/g FLW) accumulation of recombinant virus particles which displayed the chimeric rabies virus epitopes [110].

This vaccine formulation upon intraperitoneal administration triggered potent systemic neutralizing antibody reactions in mouse models and defended these mice from lethal challenge doses of rabies virus for at least 120 days. This showed the persistence of the protective immune response that is elicited through plant-derived antigens and indicated that immune memory of the rabies virus epitope could be sustainable by this immunogen.

The chimeric peptide that was displayed on the recombinant AMV particles showed higher immunogenicity than the non-conjugated peptide formulation when co-delivered with a corresponding adjuvant in mouse models. Oral delivery of spinach leaves expressing these purified AMV particles induced a strong mucosal IgA response and IgG/IgA serum immunity specific to the rabies virus.

Analogous results were also obtained in humans, showing that plant-generated chimeric cPVPs or VLPs displaying epitopes of rabies virus may yield a more economical, safe, non-parenteral vaccine alternative towards use in developing nations with a higher prevalence of the rabies disease.

## 6. Immunogenicity and Translational Outcomes of Plant Virus Expression Vectors

Plant viruses and their VLPs possess beneficial biological and physicochemical properties that enhance their potential in vaccinology. They vary in size (20–200 nm), shape, rigidity, and have well-organized structures. They represent naturally occurring nanoparticles and adjuvants, and act as immune-stimulating agents [113,114,115]. Innate immune cells can interact with them through the same pattern recognition receptors (PRRs), such as Toll-like receptors (TLRs) and C-type lectin receptors (CLRs), that detect natural viruses [115,116]. Current evidence shows that PVX, TMV, CPMV, CCMV, PhMV, AMV, and SeMV can elicit antitumor responses as in situ vaccine treatments in mice models of cancers [113,116]. The proteinaceous structure and RNA present danger signals that activate the immune system via PRRs [116,117]. For example, Affonso de Oliveira et al. revealed CPMV and PVX induce systemic, durable antitumor immunity in murine and canine models. CPMV persists longer, strongly activates Toll-like receptors, recruits macrophages, and benefits from antiviral T-cells. PVX activates TLR7 weakly [113].

Plant viruses can permeate through lymph vessel pores and are trafficked into the lymph node, and can stimulate innate immune cells, especially antigen-presenting cells (APCs) [118]. Therefore, they are highly suitable for presenting key epitopes for vaccine development in the treatment of diseases. Through genetic engineering, plant virus vectors have been endowed with capacity for display of functional peptides or proteins. They are now used to create vaccines for cancers, pandemics like H1N1, and potential biowarfare threats [7]. For example, chimeric PVX particles displaying the R9 peptide induced strong immune responses in mice with anti-R9 IgG titers of up to 1:50,000, confirming its immunogenicity and potential for hepatitis C virus epitope presentation [54]. Lozano-Sanchez and colleagues, in a 2023 study, developed a hybrid multivalent plant virus that was beaded with an anti-SARS-CoV-2 nanobody. Hybrid multivalent VNPs were generated with nanobodies against two individual epitopes of the same SARS-CoV-2 protein, mimicking the effect of a nanobody cocktail [119]. This system uses TEV and PVX coinfection. PVX supplies a second TEV CP in trans, enabling mixed assembly. Merwaiss et al. produced vaccines from plant viruses that present SARS-CoV-2 nanobodies in *N. benthamiana*, with improved neutralizing activity against pseudoviruses in cell-based infection assays [120]. In a different study, Almendárez-Rodriguez and colleagues utilized CCMV CP as a scaffold for the presentation of three SARS-CoV-2 epitopes. All three mutants were immunogenic, eliciting high IgG antibody titers that recognized the full-length S protein that was expressed recombinantly [121]. Urease subunit B (UreB) is the most effective antigen for vaccination and blocking bacterial infection. Virus-based vectors for transient expression in plants enable high levels of gene expression rapidly. Abdoli Nasab et al. studied the transient expression of UreB in rapeseed (*Brassica napus*) with a turnip mosaic virus (TuMV)-based vector. Their results indicate that rapeseed could be a rapid and economical alternative for the production of vaccine antigens in plants [122].

Plant virus vectors can be a substitute for synthetic nanoparticles or mammalian viruses for producing an mRNA vaccine delivery platform. For example, in a self-amplifying mRNA vaccine encoding Omicron, the receptor binding domain (RBD) was packaged using TMV coat proteins via in vitro assembly. It delivered and expressed RBD in mammalian cells, eliciting neutralizing IgG in mice. The TMV platform enables stable, freezer-free storage, and rapid adaptation, with potential for plant-based, localized production [123]. In another study, Royal et al. demonstrated that CoV-RBD121-NP is a stable COVID-19 vaccine candidate combining SARS-CoV-2 RBD with a TMV nanoparticle. It binds ACE2 and neutralizing antibodies, retains key epitopes, and remains potent for 12 months. In mice, it induces strong, balanced or Th1-biased immune responses and virus-neutralizing antibodies, with or without CpG adjuvant [124]. A TMV-based self-amplifying mRNA vaccine encoding a non-oncogenic mutant HPV16 E7 protein elicited E7-specific IgG and activated humoral and cellular immunity in mice. TMV coat proteins enabled stable in vitro assembly and delivery. The platform offers a promising alternative to lipid nanoparticles for mRNA vaccine development [125]. A TMV-based peptide vaccine using dissolving microneedle (MN) patches delivers PEP3 antigen transdermally. TMV-PEP3 enhances dendritic cell activation and local immune infiltration. Both MN and injection routes induce anti-PEP3 IgG and complement-dependent cytotoxicity. This approach offers a stable, immunogenic, and user-friendly platform for peptide vaccine delivery [126].

In summary, while most recombinant proteins, including interferons, are generated primarily from bacterial and mammalian cell culture-based expression systems [127], these systems all have limitations, including bacterial endotoxins, human pathogen contamination, oncogenes or prions, and insoluble inclusion body formation of recombinant proteins. Plant virus expression systems are an alternative mode of expression which do not suffer from these limitations. The inherent immunogenicity of plant viral vectors enables the development of adjuvant-free vaccines that do not require preservatives such as aluminum/mercury compounds that cause unwanted side-effects. While the oncolytic viruses gain entry into tumor cells to elicit oncolysis via infection, vectors derived from plant viruses are principally taken up by the immune cells, are non-infectious in mammals, and thereby do not directly induce cell lysis [128,129]. Plant viruses exhibit multivalency, which enhances their effectiveness through avidity effects, and also provide an adjuvant effect due to the presence of T-helper epitopes [57,129].

## 7. Recent Progress of Plant Virus Vectors in Vaccine Development

Plant virus vectors such as TMV, PVX, and AMV are production of vaccine candidates and VLPs [17]. Recent studies demonstrated robust antibody generation and protective immunity in animal models for microorganisms and cancer using them [130]. Those that are presently under clinical trials consist of Hepatitis B virus, Influenza virus, and SARS-CoV-2 vaccines, among others [131]. For example, displaying CFP10 and ESAT6 antigens of *Mycobacterium tuberculosis* (Mtb) on the surface of potyvirus-like particles (PVLPs) can induce both T- and B-cell-mediated immunity into mice [132]. Recently, co-expression of the matrix protein (M) and glycosylated protein 5 (GP5) on the surface of TMV-like particles in *Nicotiana benthamiana* resulted in induced protective immune responses against porcine reproductive and respiratory syndrome (PRRS) in animals [133]. A cucumber mosaic virus-like particle which expresses peanut allergen component Ara h 2 (VLP Peanut) was generated as a novel therapeutic approach to reduce peanut allergy (PA) through modulation of T-cells, DCs, and B-cells under in vitro conditions. AP is among the most common food allergies bereft of a favorable treatment having both efficacy and safety of use. Preliminary reports of skin reactivity used VLP Peanut in six adults having peanut allergy and was found to be well tolerated and safe in an open-label phase 1 investigation [134]. Jung et al. designed a novel PVX-based expression system to increase recombinant protein production in plants by co-expressing heterologous viral suppressors of RNA silencing (VSR). Optimized PVX-derived vectors with viral suppressors enhanced transient protein expression in *Nicotiana benthamiana*. GFP yields rose 3–4-fold, while vaccine antigens VP1 and S2 exceeded parental vectors by over 100-fold [135].

Recently, VLPs of TuMV functionalized with the staphylococcal Protein A, Z-domain through gene fusion resulted in a flexible nanoplatform that can readily bind to IgGs. For example, triple functionalization including Cy5.5 and cetuximab produced a fluorescent nanoparticle that could focus on tumor cells [136].

T-helper (Th) elicitation of Toll-like receptors (PAMPS: pathogen-associated molecular patterns), antigen organization, and antigen repetitiveness (via pathogen-associated structural patterns, PASPs) proved to be of importance in driving antibody and B-cell responses. Recently, an “immune-tag”-based vaccine strategy has been employed to combine all parameters into a highly repetitive structure. In a step-by-step fashion, the PADRE peptide AKFVAAWTLKAAA, that is an activator antigen specific to CD4+ T-cells, the third domain of the envelope protein (DV1 EDIII) of the dengue virus 1 (DENV-1), that is a major target of virus-neutralizing antibodies linked to the N-terminal fragment of the capsid protein of cucumber mosaic virus (CMV)—nCMV, and RNA were attached as a PAMP, and this elicited superior antibody responses [137]. Recently, Madapong et al. generated plant-derived vaccines harboring centralized consensus influenza virus hemagglutinin (HA-con) proteins (H1 and H3 subtypes) that were conjugated with a modified TMV nanoparticle (TMV-HA-con). Both HA-con and TMV-HA-con protein vaccines, along with adjuvants, elicited protective immune reactions against infections caused by influenza A virus [138]. Nanoparticles containing a STING agonist loaded onto the *Physalis* mottle virus internal cavity (named PhMV-STG) demonstrated augmented and prolonged interferon activity under in vitro conditions as well as extended tumor retention under in vivo conditions [139].

There exists a need to devise strategies for display of epitopes and delivery with plug-and-play technology [140]. Plug-and-play systems enable rapid plant VNPs customization for different vaccine or therapeutic targets. By reusing known backbones, these systems decrease the number of repetitive production and safety steps generally required for each new pathogen, thereby quickening large-scale manufacturing and regulatory approval simultaneously [141]. The generation of SpyCatcher-SpyTag ‘plug and play’ VLP-based systems can provide an opportunity to mitigate the requirement for specific VLP-toxin expression, thus enabling the development of a universal VLP carrier that can be decorated with antigens containing a corresponding tag [142]. For example, Liu et al. describe a new multi-antigen-based delivery system that uses a SpyCatcher/Spytag strategy in addition to self-assembled VLPs comprised of porcine circovirus type 2 (PCV2) Cap (Cap-E2 NPs), a widely utilized swine vaccine. Cap-E2 NPs formed using this strategy resulted in a more potent immune response against the classical swine fever virus (CSFV) E2 with improved antibody titer, protective cellular immune reactions, subclass distribution, and binding avidity [143].

To date, no human vaccine utilizing plant virus-like particles has been approved. Medicago’s COVID-19 vaccine was plant-derived, using *Nicotiana benthamiana* as a bioreactor, but it did not employ virus-like particles such as TMV [124]. Plants have demonstrated low protein yields; therefore, they do not produce sufficient protein for vaccine manufacturing. Additionally, many natural components within plants can make extraction and purification more difficult. Additionally, the use of genetically modified plants for vaccine production raises safety and regulatory concerns [144].

## 8. Conclusions

Plant viral transient expression approaches provide a ground-breaking means of advancing research in plant biotechnology. Their low infrastructure requirements and streamlined protocols make them ideal. The ongoing development and wider use of this technology will support future developments in synthetic biology, plant-based drugs, vaccine research, and metabolic engineering. Molecular farming is the most feasible technique for producing such products that can generate antigens and recombinant proteins in plants, including anti-cancer vaccines, for example, that can target tumor-specific antigens occurring in a person’s cancerous cells. By providing a method for efficiently making personalized vaccine candidates, it fulfills the requirement for precision in therapies like cancer vaccines. These platforms also offer a cost-effective and scalable way of manufacturing personalized vaccine candidates. This could lessen the cost of manufacturing and provide a method for personalized immunotherapy to be a strategy that can be used anywhere. However, several challenges remain. For instance, the infection process of the plant by the virus can be time-consuming [145], and plants may activate defense mechanisms such as antiviral RNA silencing to combat the infection [146]. In addition, due to size constraints, only small epitopes can be incorporated onto the virus particle surface [7]. Foreign gene inserts are often not stably maintained over time because of recombination events, which can also be host-dependent [147]. Finally, low recombinant protein yields and protein instability further limit the efficiency of these systems [135].

## Figures and Tables

**Table 1:** Examples of plant virus expression vectors used in the development of vaccines.

Plant Viral Vector	Pathogen or Protein/Epitope	Yield per Fresh Weight	References
TMV	SARS-CoV-2 spike protein (2024)	0.3/0–8 mg/g	[30]
RYMV	Promastigote Surface Antigen (PSA) protein	0.4 mg/kg	[31]
PVX	SARS-CoV-2 spike protein (2022)	80–100 μg	[32]
CPMV	Rotavirus (2020)	4.9 µg/g	[33]
TuMV	Pru p 3 (2022)	10–30 mg/100 g	[34]

Tobacco mosaic virus (TMV), rice yellow mottle virus (RYMV), potato virus X (PVX), cowpea mosaic virus (CPMV), turnip mosaic virus (TuMV).

## Data Availability

No new data were created or analyzed in this study. Data sharing is not applicable to this article.
